# Global vaccine inequities and multilateralism amid COVID-19: Reconnaissance of Global Health Diplomacy as a panacea?

**DOI:** 10.34172/hpp.2022.41

**Published:** 2022-12-31

**Authors:** Bawa Singh, Jaspal Kaur, Vijay Kumar Chattu

**Affiliations:** ^1^Department of South and Central Asian Studies, School of International Studies, Central University of Punjab, Bathinda, India; ^2^Department of Sociology, School of Social Science and Humanities, Lovely Professional University Phagwara (Punjab)-India; ^3^Department of Occupational Science & Occupational Therapy, Temerty Faculty of Medicine, University of Toronto, Toronto, ON M5G 1V7, Canada; ^4^Center for Transdisciplinary Research, Saveetha Dental College, Saveetha Institute of Medical and Technical Sciences, Saveetha University, Chennai 600077, India; ^5^Department of Community Medicine, Faculty of Medicine, Datta Meghe Institute of Medical Sciences, Wardha 442107, India

**Keywords:** COVID-19, SARS-CoV-2, Pandemic, Vaccines, Equity, Health inequities, Diplomacy, Governance, Developing countries, World Health Organization

## Abstract

**Background:** The ongoing COVID-19 pandemic has shown a crystal-clear warning that nobody will be safe until everybody is safe against the pandemic. However, how everyone is safe when the pandemic’s fat tail risks have broken every nerve of the global economy and healthcare facilities, including vaccine equity. Vaccine inequity has become one of the critical factors for millions of new infections and deaths during this pandemic. Against the backdrop of exponentially growing infected cases of COVID-19 along with vaccine in-equity, this paper will examine how multilateralism could play its role in mitigating vaccine equity through Global Health Diplomacy (GHD). Second, given the most affected developing countries’ lack of participation in multilateralism, could GHD be left as an option in the worst-case scenario?.

**Methods:** In this narrative review, a literature search was conducted in all the popular databases, such as Scopus, Web of Science, PubMed and Google search engines for the keywords in the context of developing countries and the findings are discussed in detail.

**Results:** In this multilateral world, the global governance institutions in health have been monopolized by the global North, leading to COVID-19 vaccine inequities. GHD aids health protection and public health and improves international relations. Besides, GHD facilitates a broad range of stakeholders’ commitment to collaborate in improving healthcare, achieving fair outcomes, achieving equity, and reducing poverty.

**Conclusion:** Vaccine inequity is a major challenge of the present scenario, and GHD has been partly successful in being a panacea for many countries in the global south.

## Introduction

 The ongoing black swan kind of event - the COVID-19 pandemic has coerced global leadership to rethink multilateralism and pursue Global Health Diplomacy (GHD) to combat the pandemic and one of its consequences vaccine inequity. COVID-19 has left indelible repercussions on socio-economic aspects of life and livelihood. These repercussions are more perceptible, i.e., declined economic growth; exponentially growing unemployment rates; the unavailability of basic necessities for life; inadequate healthcare services, and vaccine inequity, in particular as the by-products of the COVID-19 pandemic, a major global critical issue, even more so than a black swan event. Therefore, vaccine inequity is one of the major challenges during the pandemic peak.^1^ It is worth mentioning here that multilateralism plays an important role in controlling and fighting the pandemic through GHD. The COVID-19 pandemic has been continuously devastating lives and livelihoods across the globe. It is anticipated that the world must assess the possible impacts of the pandemic and commit to taking actions at the multilateral level, possibly to minimize the tail-end risks, ultimately leading to handling the issue of vaccine inequity. It is self-evident given the facts of loss of lives and livelihood and the multiple vulnerabilities, including the vaccine inequities, thereby putting the developing countries on the inflecting points. The pandemic created a grim situation given the doctor-to-patient relationships,^2^ lack of personal protective equipment such as face masks, gloves, and face shields as well as hospitals, beds, intensive care unit (ICU)-sleeping equipment, and oxygen to take care of the patients’ needs.^3^ Against this background, multilateralism is the best option to resolve the problems regarding the distribution of vaccines/medicines for all. The GHD at the multilateral level has been put in place to take care of the inaccessibility and affordability of vaccines and medicine to bring some fairness to be global and distributive justice against the serious blots caused by the pandemic. Targeted international support, particularly through multilateralism, is needed at the moment, along with several healthcare programs. However, it is self-evident that vaccine inequity has emerged as one of the major challenges.

 From the perspectives of global governance and policymakers, the inequity of vaccines has emerged as a global threat, along with many pestering questions. How and why has vaccine inequity been haunting and daunting developing countries? Has multilateralism taken adequate measures to resolve the vaccine inequity? Against this backdrop, it is argued that social justice is one of the major components used to focus on all the moral obligations of rich countries towards the poor. Therefore, this review is aimed to examine whether GHD can serve as a panacea to control and combat the pandemic, given the vaccine inequities.

## Materials and Methods

 For this narrative review, a literature search was conducted in all the popular databases such as Scopus, Web of Science, PubMed, and Google scholar search engines. We searched the research articles published in the English language. Emphasis was given to those articles published after the declaration of the COVID-19 pandemic in 2020 addressing vaccine inequities, multilateralism, and global health diplomacy. Relevant information on the topic was also collected from the annual reports and authentic websites of the World Health Organization (WHO), the United Nations (UN), and government ministries. Some updated information related to the context is also taken from reputed organizations such as BBC News, Reuters, and Worldometer. All the relevant articles and reports were screened for eligibility and were included if they supported the study objectives. The information about the pandemic and its impacts, the vaccine iniquities, and the aspects of multilateralism are extracted from the screened literature and the findings are presented in the results section.

## Results

 We discuss the major findings under various sub-headings: the pandemic and its multifaceted impacts, vaccine inequity, health multilateralism and its reciprocation, and GHD as a panacea.

###  Pandemic and its multifaceted impacts

 The severe acute respiratory syndrome coronavirus 2 (SARS-CoV-2) caused the pandemic of COVID-19, the current global pandemic. The pandemic originated in Wuhan at the end of December 2019 (China). Taking cognizance of the seriousness of the situation, the WHO has declared it a Public Health Emergency of International Concern (PHEIC) and a pandemic on 30 January 2020 and 11 March 2020, respectively.^4^ Currently, most countries have been undergoing multiple waves of the pandemic. It becomes crystal clear that socio-economic and geopolitical determinants of health were seriously affected, resulting in several fallouts for healthcare facilities. The pandemic has wide coverage in terms of massive losses of life and livelihood. This assertion is corroborated by the facts of infected cases, recovered cases, and deaths of 194 582 750, 176 613 887, and 4 171 672, respectively.^5^

 Additionally, it also left indelible imprints on the global economy. The world has witnessed the worst economic recession, which will not likely be recovered by 2025.^6^ Multiple factors, such as Lockdowns, the closing of industries and borders, the pandemic that caused human deaths, and unprecedented unemployment globally, contributed to the economic recession. Unemployment in most countries is anticipated to be around ten percent, and even more rates among the most severely affected COVID-19 countries.^7^ The economic recession and the oil war between Russia and Saudi Arabia over oil prices in 2020 led to a drop in oil prices, consequently resulting in the collapse of tourism, hospitality, and energy industries, directly affecting health, economy, and unemployment.^8^

 COVID-19 has also been very destructive, particularly affecting developing countries regarding food security. The World Food Program (WFP) of the United Nations cautioned on 21 April 2020 that the pandemic was anticipating a famine of biblical proportions in many parts of the world.^9^ The 2020 World Food Crises Report shows that about 55 countries have been at high risk of food security.^10^ The UN WFP has reported that a worst-case scenario will likely emerge in which “around three dozen” countries die of hunger. About 821 million people are already chronically hungry, and over a million are likely to add to this basket further.^11^ Poverty is another fallout of the pandemic. The World Bank (WB) has estimated that about 119-124 million people have been entrapped in the poverty cobweb, which is likely to rise to 143-163 million in 2021.^12^ The global healthcare system is faltering, but the same became more critical during the pandemic’s peak. Developed countries, in general, and developing countries, in particular, keep facing the lack of healthcare facilities, including vaccines. The list is very exhaustive and is not restricted to the lack of vaccines; physicians and paramedical workers, ICU beds, vaccines, medication, oxygen, equipment for life security, etc., can corroborate and support this argument.^3^ More than 192 million cases around the globe were confirmed while writing this article (25 July 2021), alongside more than 4.1 million COVID-19 deaths. Seeing its tail-end risks and loss of lives and livelihood, one of the scholars has declared the pandemic one of the most deadly in history.^13^

 It is argued that the first wave of the pandemic has not yet been controlled in several countries. At the same time, several other countries have been undergoing the second and third waves of the pandemic resulting in more vulnerabilities, fatalities, and disabilities and subverting the healthcare systems in almost all countries. The first wave caused many deaths; however, the second wave has lasting global consequences on healthcare systems. Given the trends and patterns of the cases, even the worst pandemic scenario is possible if adequate infrastructure and measures are not put in place. The economic impacts on almost all countries had left the healthcare systems of the developing countries in the lurch. Leave the manufacturing capacity of vaccines by the low-and-middle-income countries (LMICs); rather, the affordability of vaccines is more challenging. This dire situation is required a multi-faceted response through multilateralism. Now, the question emerges, how and why the vaccine inequity emerge? The next section focuses on vaccine inequity and various related perspectives.

###  Vaccine inequity

 As the global COVID-19 cases have grown exponentially, the need for vaccine development had to be accelerated by the end of 2020 since the WHO COVAX Facility (WCF) had adopted the motto “No one is safe unless everyone is safe” to underline the significance of fair distribution and immunization. This section of the paper is crucial, particularly considering how the vaccine in/equity is used to take place. Therefore, this section focuses on some questions and analysis of variables concerning vaccine inequity. The variable list included the manufacturing capacity of the developed/developing countries, accessibility, and affordability, pricing of the vaccines, distribution, etc. If these variables are not workable, then a question comes of GHD. It depends upon how efficiently GHD is pursued to equitize healthcare facilities, particularly in vaccine distribution.

 Despite having so many multilateral institutions for global health for decades, why is vaccine inequity haunting poor countries? Some obvious reasons are poverty, lack of capital, science and technology development, etc, in poor countries. On the other hand, some reasons are part of multilateralism that has failed them miserably to tackle and heal the situation. The multilateral forums have remained reactive and less proactive given the disillusionment with globalization, lack of global leadership; geopolitical tug of wars between the major power that can provide global leadership; lack of support for multilateralism, and the inadequacy of existing multilateral rules to meet new challenges and decision capacity for vaccine distribution, etc.^14^ Intellectual property rights are one of the other reasons which seriously impact vaccine equity. Besides this, manufacturing and purchase capacity are other factors determining vaccine inequities, particularly in poor countries. The most important factors, such as bilateral agreements, export restrictions, vaccine diplomacy, and manufacturing and supply chain bottlenecks, are the major factors contributing to the severe shortages of vaccines. Moreover, the world is unfair and unequal given the economy, resources, multi-dimensional and sophisticated scientific advancement, etc. However, it is argued that nobody could expect a vaccine inequity between the global rich and poor.

 Out of the total 7.8 bn population, 2.3-2.6 bn people live in LMICs countries. About 333 million in the upper middle have been living in upper-middle-income countries. On the other hand, about 972 million people reside in high-income countries. But the irony is that the poor 80% of people have access to only less than 20% of the total wealth, productive capacity, etc. In his introductory remarks, Tedros Adhanom Ghebreyesus (DG WHO) pointed out, “Vaccine equity is the problem of our time.” “We’re also failing”.^15^ To examine the vaccine inequity, one has to delve into the deployment of the COVID-19 vaccines. As of 20 July 2021, about 3.73 billion COVID-19 doses had been administered across the globe.^16^ The world’s population is at 7.8 billion, meaning that about half of the people get only one dose to date.

 The vaccine inequity becomes clearer if we compare the developed versus developing countries. Countries/region like the European Union (EU) has vaccinated about 55.8%; the UK - 68.3%; Spain - 63.5%; Canada - 70%; whereas on the other hand, the developing countries are in a very critical situation. The 16 rich countries representing only 14% of the world’s population had pre-ordered more than 53% of the potential vaccine doses.^17^ One more study^18^ reported that countries such as Australia, Canada, and Japan have only 1% of the total global COVID-19 cases. Still, the same has reserved more than 1 billion vaccine doses. Concomitantly, concerns have been raised that the rich countries are in a position to meet their vaccine demands in 2020–2021, but on the contrary, poor countries may not be able to achieve the same until 2023–2024.

**Figure 1:**
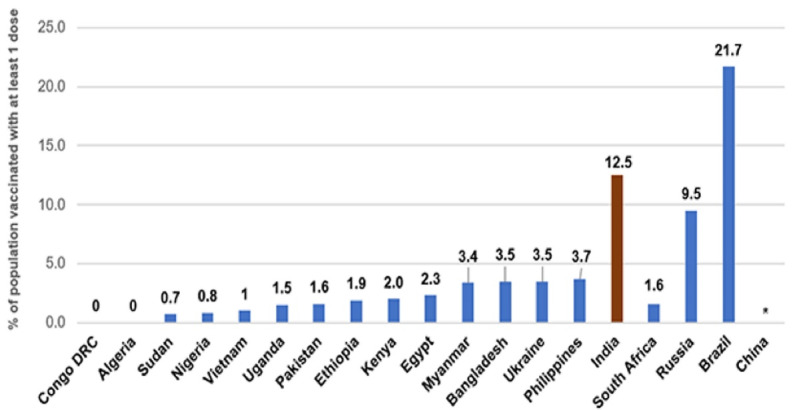
Percentage of the population vaccinated with at least one dose. Source: Bloomberg Vaccine Tracker. Accessed on 22 July 2021

 On the contrary, developing nations such as Bangladesh, Iran, Egypt, Vietnam, Ukraine, Nigeria, Ethiopia, Kenya, Angola, Uganda, Iraq, Sudan, South Sudan, DR Congo, and many countries are in very critical situations with mere vaccination coverage of 1-3% of the total population. It demonstrates that the majority of the population is bereft of the vaccination. The same is substantiated by the fact that the LMICs have been able to vaccinate only 1.1% of the population with only one dose to date.^16^ Against this scenario, Strive Masiyiwa (the African Union’s envoy for vaccine acquisition) has called it a famine situation in which “the richest guys grab the baker”.^19^ Figure 1 shows more clarity on the vaccination gaps.

 Taking cognizance of the vaccine inequity, WHO DG Tedros said, “The global distribution of vaccines still has a shocking imbalance.” He added that even there are insufficient vaccines for all health workers or risk groups in most countries, regardless of the rest of their populations.^20^ The vaccine was further hampered by many internal factors such as poverty, lack of capital, lack of science and technology, lack of infrastructure, etc. Most developing countries do not have manufacturing capacities. In manufacturing, exporting, and importing vaccines/medicines, the developing countries have not been figuring anywhere. Major pharmaceutical companies are located in developed countries. Moreover, the major exporters have mainly developed countries such as Germany ($84.7 bn), Switzerland ($71.7 bn), the United States ($49.7 bn), Belgium ($45.7 bn), and Ireland ($40 bn). Concomitantly, these countries are also the only major importers, with the US ($99.7 bn), Germany ($53.7 bn), Belgium ($36.7 bn), United Kingdom ($33.8 bn), and Switzerland ($29.3 bn).^21^ The main purpose of showing these figures here is to build the argument to highlight the vaccine monopoly. Moreover, vaccine manufacturing is monopolized by only a few countries, as shown below (Table 1).

**Table 1:** Major pharmaceutical companies

**Rank**	**Company **	**Country **	**Revenue (US$ bn)**
1	Johnson & Johnson	USA	82
2	Hoffmann-La Roche	Switzerland	63.85
3	Pfizer	USA	51.75
4	Bayer	Germany	48.02
5	Novartia	Switzerland	47.45
6	Merck & Co	USA	46.84
7	GlaxoSmithKline	UK	43.92
8	Sanofi	France	39.28
9	AbbVie	USA	33.27
10	Abbott Laboratories	USA	31.9

Source: Fact sheet (https://blog.bizvibe.com/blog/largest-pharmaceutical-companies).^21^

**Figure 2:**
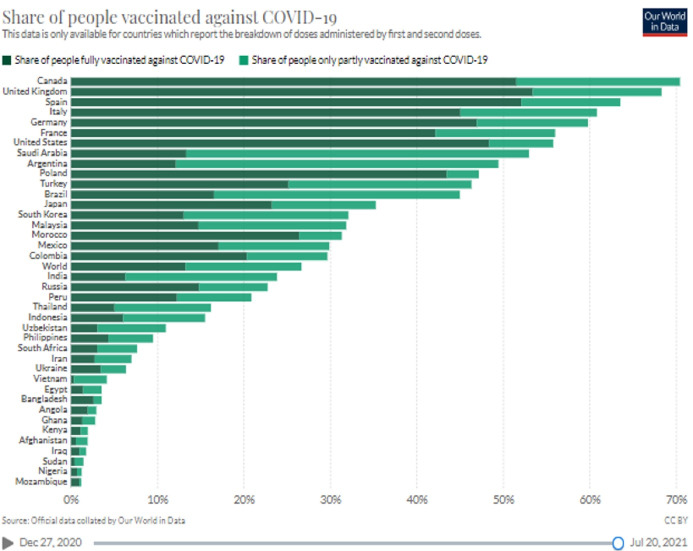
Global vaccine inequities

 Nonetheless, this is the reality, and each one is facing it now. It becomes clear that vaccine distribution between developing and developed countries continuously remained unequal even at the height of the pandemic. Therefore, it is a dire need of the moment to be immune from the virus and a large proportion of the population to end the pandemic. Administering vaccine is the safest way to protect humanity. However, despite the several faults, it is highly appreciable that several research teams from developed countries took up this challenge and developed and manufactured vaccines against SARS-CoV-2 within one year of the onset of the COVID-19 pandemic. The vaccine inequity becomes clear from the Figure 2.

###  Multilateralism and Global Health Diplomacy: marriage of complementarities 

 The origination of multilateralism can be seen no sooner than later signing of the UN Charter in 1945. During the celebrations of the 75th anniversary of the UN amidst the pandemic, the demand was raised for collective endeavors and shared responsibilities in promoting multilateralism.^22^ de Wijk et al^23^ argued that multilateralism is defined and characterized by such conditions-the inter-governmental relations; the politics of power undermined by building a stable relationship for mutual gains; security cooperation; open trade, etc. The ongoing pandemic and the rising COVID-19 waves have again highlighted the need for the potential role of liberal approaches-multilateralism as one of the best options to handle the ongoing world health emergency crisis.Keohane^24^ has argued that the role of multilateralism and intergovernmental organizations that treat the state as one of the rational actors within the international political systems is an essential element of this approach. Multilateralism is considered one of liberal institutionalism’s diplomatic approaches. However, Narlikar has argued that in the present context, the multilateral forums have been at their lowest ebb because of disillusionment with globalization, lackluster narratives in support of multilateralism, and the inadequacy of existing multilateral rules to meet new challenges need surge similar to the pandemic waves.^14^

 The COVID-19 pandemic, a global health concern, cannot be controlled at the local/national/regional levels. Instead, it requires a global response through multilateralism.

 Some scholars have argued that multilateralism is governance by multiple actors to address shared common problems. Some common principles are guiding principles among the parties, including agreed rules/principles of behavior. Multilateralism is generally comprised of membership.^25^ At the same time, among the various types of multilateralism, one is universal multilateralism, including all states, for example, the UN, WHO, World Trade Organization (WTO), etc. In the present context, it is the ongoing global health crisis, wherein WHO’s role becomes critical to analyze and examine how it has dealt with the situation employing GHD. In multilateralism, all the major multilateral organizations are considered part of the UN, and the WHO stands at the top hierarchically in multilateralism concerning health issues and decisions. Health multilateralism has an important role in health issues. The health issues are being looked after through the GHD, an important part of multilateralism. What is GHD? how is it linked with multilateralism, and how it plays a role in dealing with such health emergencies?

**Figure 3:**
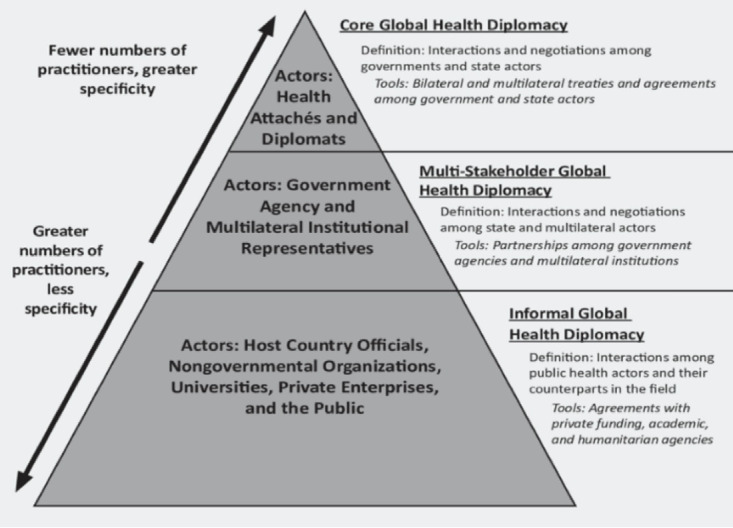
Various forms of Global Health Diplomacy as per the actors involved.^28^

 According to the WHO EMRO, GHD is used to strengthen health protection and public health, improve international relations, and a broad range of stakeholders’ commitment to collaborate in improving healthcare and achieving fair outcomes to help reduce poverty and achieve equity.^26^ According to Kickbusch and Kökény, four factors have contributed to the acceptance and universalization of GHD. One, because of having soft power importance, it is a tool for improving bilateral and multilateral relations; two, the realm of health diplomacy is expanding, including WHO is shaping the global policy in the sphere of health and its determinants; third, globalization has heightened the need for health diplomacy, leading to binding and non-binding agreements. Fourthly, health diplomats’ role in making agreements and negotiations is relevant.^27^ As depicted below (Figure 3), the explanation of the concept of GHD becomes clearer through the involvement of various stakeholders at different levels and their overall influence.^28^

###  Global Health Diplomacy as a panacea

 This section highlights the role played by GHD in addressing the vaccine equity gaps and the successful negotiations with multiple stakeholders in the multilateral global landscape. The review has identified core strategies and initiatives that have resulted in the scaling up of vaccine coverage through establishing the COVAX facility, which is discussed in detail.

####  Vaccine equity

 Soon after the pandemic outbreak, multilateral institutions played their role constructively. The various campaigns, missions, summits, and virtual meetings have been organized by multilateral institutions such as the UN, WHO, WB, IMF, WTO, G7, G20, SCO, Quad, etc, and reiterated their commitment to vaccine distribution to the needy countries, taken as a major step in the direction of vaccine equity. Since WHO is at the top of the multilateral hierarchy regarding technical expertise in issues related to global health governance, its role needs to be discussed accordingly. On January 1, 2020, the WHO established an Incident Management Support Team (IMST), informed all the member countries about the outbreak and provided advice on how to respond. Rather, COVID-19 has been declared a PHEIC and urged all countries to take a “call to action” to provide testing, protective equipment, and medical supplies for low-income countries to help them combat and control the crisis.

 The WHO DG and Executive Director of the WHO Health Emergencies Programme have held 120 media briefings concerning the pandemic issues. About 38 Member State briefings and information sessions were conducted. WHO has convened several international expert networks for several health-related topics, including the research and development for diagnostics, therapeutics, and vaccines through frequent teleconferences since the inception of the pandemic. In these networks, thousands of medical and public health professionals, including top scientists across the globe, were engaged. The OpenWHO platform was launched to start 149 courses to heighten the support for the COVID-19 response. On 1 January 2020, the WHO activated its IMST to coordinate activities and responses across the three levels of the WHO (Headquarters, Regional, and Country) for public health emergencies. The Global Outbreak Alert and Response Network was launched on 3 January 2020, which gives scientific forecasts based on pandemic data. The DG WHO addressed the G20 Leaders’ Summit and called to ensure COVID-19 vaccines as global public goods. Concomitantly, he urged the countries to implement the International Health Regulations 2005, address the vulnerabilities and inequalities, and bridge the financing gaps of Access to COVID-19 Tools Accelerator (ACT-A). The WHO and WB established the Global Preparedness Monitoring Board to track the global preparedness for health emergencies and called for an immediate arrangement and injection of US$ 8 billion to prioritize the delivery of vaccines to the most vulnerable countries.

####  WHO COVAX

 The WCF has been working day and night on the motto, “No one is safe unless everyone is safe,” underlining the need for equitable vaccination. For this, WHO, in collaboration with Global Alliance for Vaccines and Immunization (GAVI), the Coalition for Epidemic Preparedness Innovations (CEPI), and ACT-A, have played a very constructive role. About 165 countries join COVAX accounting for 60% of the world’s population.^29^ These partners provide equitable access to COVID-19 tests, therapies, and vaccines for low-income countries.^30^ To achieve vaccine equity, these partners need a fund of US$ 6.8 billion in 2021. Of this, about US$ 800 million for vaccine research and development, US$ 4.6 billion for the COVAX Advance Market Commitment, and US$ 1.4 billion would be allocated for delivery support. It is said that under the same, COVID-19 vaccines would be provided to 100 LMICs.^31^ The WCF has also signed agreements with several vaccine manufacturers to supply 1.3 billion doses for 92 LMICs during the first half of 2021.^32^ By the end of 2021, the goal of this initiative is to provide 2 billion doses, which could be considered sufficient to protect high-risk and vulnerable individuals and frontline healthcare workers.

 The UK organized the Global Vaccine Summit (GVS) in 2021, wherein representatives from the UN agencies, 50 countries, and civil societies participated in the summit. This summit aimed to seek funding for equitable and fair vaccinations to protect humanity from ongoing and future pandemics. The GVS intended to raise funds to US$ 7.4 million to immunize 300 million children in the world’s poorest countries by 2025. During the GVS 2021, the participants reiterated the need to focus on the Global Vaccine Action Plan (GVAP) 2011-2020. The GVAP is a framework to prevent millions of deaths by 2020 through more equitable access to existing vaccines for people in all communities.^33^

####  Coalition for Epidemic Preparedness Innovations 

 The CEPI has an important role in vaccine equity. In April 2020, the CEPI reported that “Strong international coordination and cooperation between vaccine developers, regulators, policymakers, funders, public health bodies, and governments would be needed to ensure that promising late-stage vaccine candidates can be manufactured in sufficient quantities and equitably supplied to all affected areas, particularly low-resource regions.” For the same purpose, the WHO and CEPI are urging funds to ensure vaccine equity for COVID-19.^34^ The collaboration of the CEPI with the WHO ensures the allocation of COVID-19 vaccines for frontline healthcare workers, the elderly, and impoverished people. The CEPI has also revised its equitable access policy concerning funding. The key changes have been implemented, as vaccine prices may be set as low as feasible for countries/territories affected by a pandemic for which CEPI funding was used to develop vaccines. The vaccine production technology must be shared with the CEPI to assume the responsibility for vaccine development if the company stops producing it. Concomitantly, the CEPI would own the intellectual property rights to the promising vaccines, enjoy the financial rewards of CEPI-sponsored vaccine development, and make the reinvestment to offer global public health benefits and data openness.^35^ During the first half of 2020, the WHO, with CEP, the Bill & Melinda Gates Foundation, and the GAVI, created a vaccine development fund and distribution of vaccinations for developing countries. These institutions have raised over US$ 20 billion in funds for the same.^36^ Some significant initiatives in the health equity direction include the Global Health Security Agenda, universal health coverage, Sustainable Development Goals, etc. The GHD played a significant role on the part of the allied agencies in general and the WHO in particular.

## Discussion

 The UN has been on the frontline to fight against the pandemic since its first outbreak. It has responded by pushing up the development, manufacture, and equitable distribution of COVID-19 diagnostics, treatments, and vaccines. The second response is to make a wide-ranging effort to safeguard lives and livelihoods by addressing the devastating short and long terms socio-economic, humanitarian, and human rights aspects of a pandemic concerning that were hit hardest. Under this, the emphasis has been given to lifesaving by ensuring access to essential medical services and supply chains and putting human rights first. These objectives have been achieved by offering immediate humanitarian aid to the most vulnerable 63 nations through the Global Humanitarian Response Plan (GHRP) and support to over 120 countries for an immediate socio-economic response guided by the UN development system framework. Globally, the GHRP has comprised the policy agenda outlined in a series of policy briefs and strong advocacy for aid to developing nations, such as a debt freeze, debt restructuring, and increased help from international financial institutions. The third response is not directly related to COVID-19 but resolves the allied issues to address underlying fragilities and identifies chances for revolutionary change toward a more just, egalitarian, and resilient society and economies, leading to a better post-COVID-19 future. Overall, the entire UN system has been mobilized behind the WHO-led health response for the distribution of medical supplies; training of health workers, testing and tracing capacities; preventing the spread of the virus, particularly among vulnerable populations, etc.

**Figure 4:**
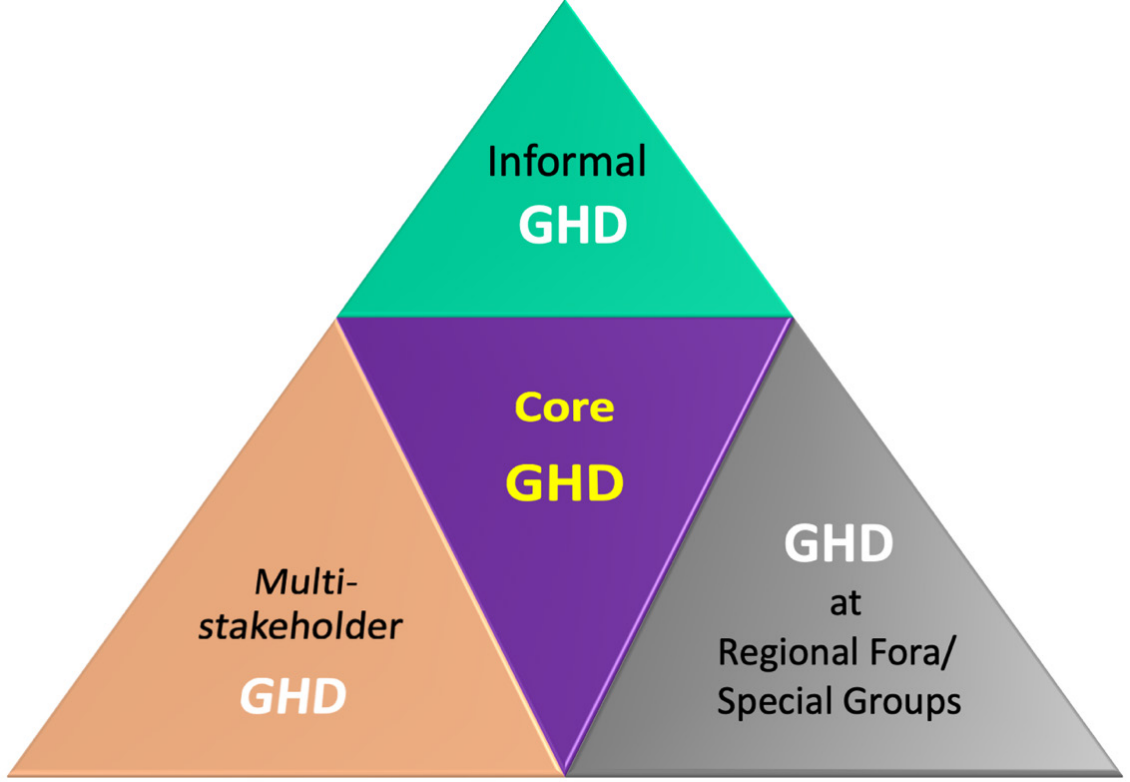
Engagement of various stakeholders in global health diplomacy to address the COVID-19 pandemic

 More recently, during this pandemic, we have observed that GHD is taking place at all venues since ‘health’ has taken over the overall priority in the global agenda due to the global impacts of COVID-19. This is more so during the regional meets (ASEAN, SAARC, CARICOM, EU, AU, etc), special group summits (G7, G20, BRICS) along with the traditional international multilateral settings (WHO, WTO, UN, etc). The core GHD talks dedicated to health issues, challenges, and emergencies happen at multilateral institutions like WHO and UN during the World Health Assembly (WHA), UN General Assembly, and Human Rights Council meetings **(**Figure 4). The GHD helps conceptualize and realize international treaties, conventions, accords, and agreements.

 The regional organizations have also come forward and shown their commitment toward the poor/member countries. During the G7 Summit in Cornwall, England (11-13 June 2021), developing a response to the COVID-19 pandemic has remained one of the main agendas. A call was taken to work on a global approach for ensuring vaccine equity to prevent future pandemics, along with donating a billion COVID-19 vaccine doses to poorer countries.^37^ President Biden and President Macron had expressed their support for the TRIPS waiver.^38^The G20 has also reiterated its commitment to extend support to bring the pandemic under control. Equitable vaccination, in combination with appropriate other public health measures, remains a group’s top priority. It reiterated its support for all collaborative activities, particularly the ACT-A, to expand COVID-19 immunization as a worldwide public good. During the Summit, the group also underlined the need to bridge the ACT-A funding gap and promote the worldwide sharing of safe, effective, high-quality, and affordable vaccine doses.^39^

 Out of Quad Summit 2021, the call for launching the Quad Vaccine Partnership has taken. Quad partners committed to working collaboratively to achieve expanded manufacturing of safe and effective COVID-19 vaccines. Priority will be given to expanding vaccine manufacturing capacity by addressing the financing and logistical demands for producing, procuring, and delivering safe and effective vaccines. The US Development Finance Corporation, Japan International Cooperation Agency, and Japan Bank of International Cooperation committed to working together to put into use the shared tools and expertise. The QUAD partner would extend finance to provide 1 billion doses of COVID-19 vaccines by the end of 2022.^39^ Australia committed to contributing US$ 77 million for the provision of vaccines with a focus on Southeast Asia, along with a commitment of US$ 407 million for nine Pacific Island countries’ health security in Southeast Asia. Japan to support vaccination programs of developing countries with the provision of $41 million and new concessional loans and support the COVAX. The US to further boost vaccination capability with at least $100 million for regional immunization. India has pursued aggressive health diplomacy and provided vaccines to more than 100 countries; however, the same was dropped given its second-wave challenges.^16^

 During the SCO Summit 2020, the SCO Member States’ underlined the need to orientate a common vision to the face of pandemics by focusing on the free and open sharing of the benefits of medical research and development and developing adaptive, responsive, and humane health systems. Under the vision of global prosperity, they stood for eliminating the consequences of the COVID-19 pandemic along with countering counterfeit medicines and the development of medical tourism, etc.^40^ South Asian Association for Regional Cooperation (SAARC) Coronavirus Emergency Fund was established during the virtual conference. The goal of the program is to mitigate the pandemic crisis in the South Asian region. India had provided around 65.5 million doses of COVID-19 vaccines to 95 countries by late March 2021.^41^

 Since the outbreak of COVID-19, the European Union has aligned with several multilateral institutions and pursued GHD. The EU is one of the founders who established the ACT-A initiative and the COVAX Facility, the worldwide initiatives to bring governments and manufacturers on one platform to ensure the COVID-19 vaccinations reach those in urgent need of the vaccines. It emphasized equitable access to the COVID-19 vaccines. The EU has organized the Coronavirus Global Response Pledging Conference (4 May), which sought to ensure that everyone had equal access to safe and effective COVID-19 vaccines. The EU held a pledge marathon by which it raised €15.9 billion to develop and deploy vaccinations, testing, and treatments.^42^ By the end of 2021, the EU would invest up to 3 billion to help acquire 1.8 billion doses of vaccinations for the LMICs.^42^

 The European Commission launched a new campaign, ‘Global Goal: Unite for Our Future,’ culminating in a Global Pledging Summit (27 June)^43^. It gave more than $1.5 billion as new grants and $5.4 bn in loans and guarantees amounting to $ 6.9 billion for providing equitable access to COVID-19 tests and treatments in general and equity access to vaccines in the poorest countries.^44^ At a virtual fundraising event on June 27, 2020, European Commission President Ursula von der Leyen announced that the European Union would provide an additional €4.9 billion to make coronavirus vaccines, diagnostic tests, and treatments available around the world.^45^ Concerning the multilateral front, the EU extended its support to the WHO’s global public health response, such as the proposal for COVID-19 resolution at the WHA in May 2020.^46^

## Conclusion

 The COVID-19 pandemic has left indelible multi-dimensional imprints in terms of social, economic, political, and geopolitical, including the dilapidation and paralysis of healthcare systems across the globe. It has put human lives and livelihoods at the inflection point, thereby turning the world unfair and unequal in general and concerning vaccine inequity in particular. However, the moral obligations of global justice, peace, and solidarity mandate that rich countries help the poor countries to mitigate these impacts alongside combat and control the pandemic by pursuing the GHD through the multilateral system. Beyond an iota of doubt, on the part of the multilateral institutions, the GHD pursued very proactively. However, the scale and substance of the pandemic, whatever efforts have been made until now, have proved ineffective given the vaccine monopoly by the rich countries. The rich countries pre-ordered more than their requirement and even vaccinated their population an average of 60%, whereas the poor countries had covered by vaccination only 1.1% of their population. This means that despite the GHD in place, vaccine equity remained a Gordian knot and conundrum for the present generation. The vaccine inequity is likely to remain a more critical challenge when the third wave is anticipated, by which the worst scenario is yet to come. Lastly, we conclude the paper by arguing that vaccine inequity is the major challenge of the present scenario, and GHD has been partly successful in being a panacea for handling vaccine inequities. Therefore with solidarity in mind, the efforts of GHD should not lose its focus to address vaccine equity gaps in the developing world.

## Author Contributions

**Conceptualization:** Bawa Singh, Vijay Kumar Chattu.

**Methodology:** Bawa Singh, Vijay Kumar Chattu.

**Investigation:** Bawa Singh, Jaspal Kaur.

**Resources:** Bawa Singh.

**Writing—original draft preparation:** Bawa Singh, Jaspal Kaur and Vijay Kumar Chattu.

**Manuscript—review and editing:** Vijay Kumar Chattu.

**Supervision:** Vijay Kumar Chattu.

**Project administration:** Bawa Singh, Vijay Kumar Chattu.

 All authors have read and agreed to the final version of the manuscript.

## Funding

 This research received no external funding.

## Ethical Approval

 Not applicable.

## Competing Interests

 Vijay Kumar Chattu is one of the editorial board members of *Health Promotion Perspectives*. Other authors declare no competing interests.
